# The Positive Effect of pro‐Environmental Behavior on Eudaimonic Well‐Being in Young Adults: A Daily Diary Study Using the Within‐Person Encouragement Design

**DOI:** 10.1111/jopy.13021

**Published:** 2025-03-20

**Authors:** Silvia Caldaroni, Maria Gerbino, Florian Schmiedek, Andreas B. Neubauer, Lucia Manfredi, Fulvio Gregori, Concetta Pastorelli, Giuseppe Corbelli, Antonio Zuffianò

**Affiliations:** ^1^ Department of Psychology Sapienza, University of Rome Rome Italy; ^2^ IDeA Center for Research on Individual Development and Adaptive Education of Children at Risk Leibniz Institute for Research and Information in Education Frankfurt am Main Germany; ^3^ Institute of Psychology, Goethe University Frankfurt Frankfurt am Main Germany; ^4^ Institute of Psychology, RWTH Aachen University Aachen Germany

**Keywords:** dynamic structural equation modeling, eudaimonic well‐being, instrumental variable estimation, pro‐environmental behavior, within‐person encouragement design

## Abstract

**Introduction:**

Existing literature has highlighted the relevance of Pro‐environmental behaviors (PEBs)—actions intended to benefit the environment—to Eudaimonic well‐being (EWB, i.e., meaning in life and connectedness to others). However, most research has focused on stable individual differences and utilized cross‐sectional designs, giving limited attention to the momentary fluctuations of PEBs within individuals. This study aimed to investigate the daily impact of PEBs on EWB from a causal perspective, examining whether manipulating daily PEBs would result in higher levels of EWB on those days.

**Method:**

We adopted the Within‐Person Encouragement Design, an experimental approach employing instrumental variable estimation, in a Dynamic Structural Equation Modeling framework. Participants were 63 Italian young adults assessed over 21 days and who received 11 randomized encouragements (i.e., “Today we ask you to implement more pro‐environmental actions than you would normally enact on a typical day”).

**Results:**

A significant positive adherence effect of the encouragement on PEB, and a significant positive treatment effect of PEB on EWB was found.

**Conclusion:**

These findings are promising for advancing successful behavioral interventions designed to encourage daily PEBs in younger generations and highlight the importance of PEBs for experiencing a more meaningful life and enhanced connectedness with others.

## 1 Introduction

Given that individuals can play a significant role in addressing the challenges posed by environmental degradation and climate change (Steg et al. 2014; Syropoulos and Markowitz 2024), significant attention has been devoted to studying Pro‐environmental behaviors (PEBs)—actions that benefit the environment or minimize harm to it (Steg and Vlek 2009). Through engagement in PEBs, individuals not only contribute to environmental preservation but also enhance their personal well‐being (e.g., Brown and Kasser 2005; Prati et al. 2017; Shin et al. 2022; Suárez‐Varela et al. 2016; Zawadzki et al. 2020). This positive effect can be immediate; namely, it can occur on a daily basis (e.g., Bissing‐Olson et al. 2013; Wray‐Lake et al. 2019).

Although PEBs oftentimes require a certain degree of effort (Nielsen 2017) as they can be uncomfortable or inconvenient, previous literature has found that they are associated with experiencing positive emotions and feeling good about themselves (Venhoeven et al. 2020). The relationship between PEB and well‐being has been investigated in terms of two distinct dimensions of well‐being known as hedonic and eudaimonic (Shin et al. 2022). The former focuses on pleasure as a primary driver of human behavior and is characterized by the presence of positive emotions and life satisfaction (Diener et al. 1999). The latter, Eudaimonic Well‐Being (EWB), refers to self‐realization and living a fulfilling life (Ryan and Deci 2001; Deci and Ryan 2008).

Existing research on the association between PEB and well‐being presents substantial gaps that the present study aims to address. Firstly, it has primarily focused on the hedonic aspects of well‐being (Netuveli and Watts 2020). However, a more thorough investigation concerning EWB can be relevant to understanding how PEB can promote a deeper experience of well‐being (Venhoeven et al. 2013), namely cultivating one's best qualities through engagement in activities that are aligned with one's overarching goals (Huta and Ryan 2010). Specifically, among the relevant components of EWB (Ryff and Singer 2008) that have been found to be positively associated with PEB (Venhoeven et al. 2020; Prati et al. 2017) are meaning in life, which involves perceiving life as having sense and direction, and closeness to others (Deci and Ryan 2008; Huta and Ryan 2010), which entails feeling connected to others and nurturing social relationships that foster care and personal development (Ryff and Singer 2008).

Secondly, the association between PEBs and EWB has predominantly been examined in terms of stable interindividual (between‐person) differences, primarily through cross‐sectional studies. However, this investigation should be accompanied by a deeper examination of intraindividual (within‐person) processes to give a more comprehensive view of individual functioning in terms of dynamic changes in behaviors and experiences (Kuper et al. 2021) that occur over short time periods (Hamaker et al. 2018). The use of intensive longitudinal data—that is, the collection of multiple measurement occasions over a brief observation period (McNeish and Hamaker 2020) through Experience Sampling Methods (ESM; Hektner et al. 2007)—is particularly suitable for exploring the influence of PEB on EWB in everyday life, as well as representing an ecologically valid approach (Hamaker et al. 2018).

Therefore, in the present study, we aimed to explore the impact of daily PEBs on EWB, specifically investigating whether engaging in more PEBs than usual on a given day could lead to higher levels of EWB than usual on the same day. Moreover, to draw causal inferences (Hamaker and Wichers 2017; Rohrer and Murayama 2023) on such an individual experience of enhanced well‐being derived from PEB performance, we manipulated PEBs by providing participants with random daily encouragements to engage in more PEBs than they typically do. Employing the Within‐Person Encouragement Design (WPED; Schmiedek and Neubauer 2020) combined with Dynamic Structural Equation Modeling (DSEM; Hamaker et al. 2018), we analyzed daily diaries (Bolger et al. 2003) collected from a sample of Italian young adults followed over a period of 21 days.

### 1.1 Pro‐Environmental Behaviors and Eudaimonic Well‐Being

PEB is an umbrella term that refers to actions that either directly benefit the environment, such as recycling and resource conservation (e.g., energy and water), or minimize the negative impact on it, namely limitation of plastic consumption (Lange and Dewitte 2019; Lutz et al. 2023; Steg and Vlek 2009; Stern 2000). People can contribute to environmental preservation also indirectly by motivating other people (e.g., family and friends) to engage in PEBs (Hanss and Böhm 2010). As PEBs often entail some level of inconvenience or difficulty, performing them can be personally challenging (Nielsen 2017; Nielsen and Hofmann 2021). Specifically, PEBs can be costly (Klein et al. 2022) in terms of both material and psychological resources, as they can be time‐consuming, expensive, or unpleasant (Steg et al. 2014), and the resulting benefits are often long‐term, indirect, or external to the individual (De Groot and Steg 2009; Klein et al. 2022). Nevertheless, research has found that PEBs can improve individuals' EWB (Venhoeven et al. 2013), specifically by evoking a sense of meaning in life and closeness to others (Deci and Ryan 2008). From a conceptual perspective, engaging in PEBs might foster EWB by satisfying the core human needs of agency and communion (Bakan 1966). Regarding agency, as highlighted by Bandura (1989, 2001), individuals can proactively shape their social and natural environments by exercising personal agency (Caprara and Steca 2007; Sawitri et al. 2015). This involves setting goals, monitoring behaviors that reflect personal values, self‐evaluating actions, and learning from personal and others' experiences. By doing so, individuals contribute not only to environmental protection but also to their personal adaptation and development (Bandura 2001). In this line, a sense of responsibility toward the natural environment can motivate individuals to take actions aimed at its preservation (Cuadrado et al. 2023; Sawitri et al. 2015). Moreover, given that people are oriented to personal development (Huta and Ryan 2010), engaging in PEBs represents a way to express one's best capacities, and this is particularly true when such actions are deliberately chosen and imply challenging personal boundaries, namely when individuals exert personal efforts to engage in PEBs, they are likely to experience enhanced EWB (Venhoeven et al. 2013). In this context, choosing to do good rather than harm can make people feel good about themselves (Zawadzki et al. 2020). In terms of communion, which refers to a sense of belonging and the cultivation of positive, meaningful social relationships (Bakan 1966), PEBs, as other‐oriented behaviors, can foster the perception that an individual is actively pursuing beneficial goals for the collective and contributing to social welfare (Prati et al. 2017). These aspects reflect the desire to belong to a community and be valued within society (Mac Donald and Staats 2022).

A literature review shows that prior studies investigated the association of PEBs with distinct facets of eudaimonia, like eudaimonic values (Chan et al. 2016), eudaimonic happiness (Zawadzki et al. 2020), closeness to others (Prati et al. 2017), and a sense of meaning in life. Considering the latter, a recent meta‐analysis conducted by Zawadzki et al. (2020) revealed that the positive association between PEBs and well‐being was stronger for those aspects that reflected a sense of meaning, such as purchase behaviors that are deliberately chosen and eudaimonic happiness that reflects individuals' perception of their lives being meaningful. In line with these findings, another study (Venhoeven et al. 2020) investigating the association between meaning attribution and positive emotions regarding PEB performance revealed that individuals engaging in PEBs feel good particularly when such experiences are personally relevant. Other researchers (e.g., Chan et al. 2016) have examined the role of eudaimonia as a human value that enhances human‐nature connections, thereby contributing to a meaningful life. Finally, literature on sustainable consumption also evidenced that this type of PEB fosters meaning in life (e.g., Carrero et al. 2020; Ganglmair‐Wooliscroft and Wooliscroft 2016). Concerning research on PEB and closeness to other people (Deci and Ryan 2008), a long‐term longitudinal study on energy conservation by Prati et al. (2017) found that PEB positively predicted subsequent social well‐being, a form of EWB referred to as nurturing positive relationships. In turn, Mac Donald and Staats (2022) evidenced that the desire to belong to a community and be appreciated by other people can motivate engagement in various PEBs that reflect an orientation toward the collective welfare, like talking with others about environmental issues or being involved in pro‐environmental initiatives.

### 1.2 The Present Study

Existing literature has shown the relevance of PEBs to EWB by focusing on stable differences between individuals, mostly employing cross‐sectional designs (Prinzing 2024; Wray‐Lake et al. 2019) that do not differentiate between interindividual (between) and intraindividual (within) variability (Hamaker et al. 2018). Furthermore, rather than focusing on EWB, existing studies employing ESM mostly examined well‐being outcomes related to hedonism, such as positive and negative affect and life satisfaction (e.g., Bissing‐Olson et al. 2016, 2013; Wray‐Lake et al. 2019) and to our knowledge, only one measured well‐being including meaning in life (Prinzing 2024).

While these studies show promising results, limited attention has been paid to exploring state‐like, momentary fluctuations in PEBs within individuals. Such an investigation may provide a more granular understanding of the dynamic, intrapsychological processes involved in daily PEB (Richter and Hunecke 2022) as well as their impact on momentary EWB in everyday life. For example, regarding prosocial behavior, of which PEB can be considered a form (Otto et al. 2021), a recent study investigating the reciprocal relationship between momentary fluctuations in prosocial behavior and EWB found that changes in prosocial behavior significantly impacted EWB in two samples of adults and adolescents (Gregori et al. 2024). To better capture these dynamics, intensive longitudinal data collected through ESM, such as daily diaries (Bolger et al. 2003), may offer advantages in terms of reduced recall bias and improved ecological validity (Hamaker and Wichers 2017), as well as be a means to assess more precisely the effectiveness of interventions aimed at promoting PEBs (Lange and Dewitte 2019). More importantly, these methods can provide key insights into how the positive effects of PEBs on EWB unfold over time (Rohrer and Murayama 2023; Voelkle et al. 2018), while accounting for stable between‐person differences (Hamaker et al. 2018). Despite these advantages, the observational nature of intensive longitudinal data obtained through ESM prevents drawing causal conclusions due to the presence of uncontrolled factors that can explain the influence of daily PEBs on EWB (Antonakis et al. 2010; Hamaker and Wichers 2017; Rohrer and Murayama 2023). A way to overcome such a limitation is integrating within‐person data with experimental methods, namely manipulating daily PEBs as the focal explanatory variable (Schmiedek and Neubauer 2020).

In the present study, we aimed to address these gaps by examining the putative causal effect of PEBs on EWB in the daily lives of young adults. We combined intensive longitudinal data with an experimental approach that incorporates a within‐person manipulation (as in micro‐randomized trials; see Klasnja et al. 2015) with the use of encouragements (Bradlow 1998; Powers and Swinton 1984). Specifically, we implemented the WPED introduced by Schmiedek and Neubauer (2020), which integrates within‐person manipulations of a treatment variable (in our case, PEBs) and random encouragements to evaluate the putative causal effect of the treatment on a third variable, the outcome (i.e., EWB). The aim was to assess not only the changes in behavior but also the enhancement of a distal outcome, specifically well‐being (Reichert et al. 2020).

In detail, we employed daily diary data collected among Italian young adults to assess their levels of PEBs and EWB once a day over 21 days. Over 11 non‐consecutive days, participants were encouraged, “to engage in more pro‐environmental behaviors than usual,” while on other days they were allowed to behave normally. Such an encouragement functioned as a random manipulation of PEBs across occasions, as it was activated randomly on a subset of occasions and deactivated on the rest (Schmiedek and Neubauer 2020). Via the WPED, we were able to evaluate what happened in terms of well‐being when a person showed higher engagement in PEBs due to encouragement compared to when the same person did not show the same behavior. To our knowledge, only a study by Prinzing (2024) employed encouragements to investigate the effect of PEBs on young adults' well‐being. However, the manipulation in this study was at the between‐person level: participants were randomly assigned to one of three experimental conditions including a pro‐environmental one in which they were prompted “to do three good things for the planet” the next day.

Regarding the period of life examined (young adulthood), investigating the impact of PEB on EWB during this life stage can offer valuable insights into how these behaviors represent adaptive responses to environmental challenges (Bøhlerengen and Wiium 2022; Pereira and Freire 2021). Such actions are crucial during a pivotal life stage often marked by challenges, including the transition into adulthood (Wray‐Lake et al. 2019) and concerns about climate vulnerability (Ardoin et al. 2022). A recent survey by the European Investment Bank (2024) indicates that young Italians are particularly informed about climate change, with a substantial majority of respondents (around 72%) believing that their actions can contribute to addressing climate challenges. This reflects a broader trend of awareness and concern among younger generations, who feel a strong sense of personal responsibility regarding climate issues.

Concerning the research hypotheses, drawing from prior literature (e.g., Bissing‐Olson et al. 2013; Venhoeven et al. 2013; Zawadzki et al. 2020), we expected that manipulating PEBs—that is, prompting on a given day—would result in higher than usual levels of PEBs (i.e., encouragement effect), which in turn would lead to enhanced EWB on that day (i.e., treatment effect). Additionally, we hypothesized a positive persistence (i.e., carry‐over effects) of both PEBs and EWB on consecutive days, namely, higher than usual levels of PEBs on a day would predict higher than usual levels of such behaviors the following day (more than usual). Similarly, positive deviations from one's average levels of EWB on a day would predict positive deviations of EWB the next day. These potential carry‐over effects were captured by autoregressive effects in the statistical model.

From a methodological perspective, the WPED allowed us to draw causal conclusions regarding the impact of PEB on EWB by isolating the exogenous portion of PEB variability and testing its effect on EWB, overcoming the so‐called endogeneity issues deriving from potential time‐varying confounders (Rohrer and Murayama 2023), as well as the ethical and practical challenges of experimental studies deriving from assigning participants to various treatment conditions (Bradlow 1998). Finally, employing DSEM (Hamaker et al. 2018) to analyze intensive longitudinal data enabled us to disentangle momentary within‐person (state‐like) changes from stable between‐person (trait‐like) differences.

No hypotheses were preregistered for this study.

## 2 Method

### 2.1 Participants and Procedure

The sample was composed of 63 Italian young adults (ages ranged from 19 to 35 years, M_age_ = 25.4, SD = 3.5, 68% females) and was part of a larger longitudinal research study aimed at investigating the role of prosocial behavior, emotional regulation, and pro‐environmental behavior in fostering young adults' well‐being in their daily life, and which received ethical approval by the author's institution [identity withheld for blind peer review]. Following the recommendations of Schmiedek and Neubauer (2020) for intensive longitudinal designs, we aimed to collect a total number of observations (persons × occasions) in the range of thousands to ensure adequate statistical power for detecting the effect of a treatment implemented via encouragements. Additionally, based on the recommendations of Maas and Hox (2005) for nested data, we aimed to recruit more than 50 participants (i.e., level 2 units).

Participants were compensated with a bookshop gift voucher for a maximum value of 25 euros as appreciation for their time. The study was structured as follows. Before the study began, participants were asked to download the experience sampling app (i.e., Metricwire) on their smartphone to receive encouragement and to fill out the daily questionnaires. The participants were informed that each day they would report on a series of psychological aspects, including their well‐being, positive and negative affect, pro‐social behaviors, and PEBs. They were also informed that on some days, they would receive encouragement to engage in more PEBs than usual, but they were free to choose whether or not to follow the prompt, with no consequences for their decision. Additionally, on days without encouragement, participants were instructed to behave as they normally would in their daily lives.

Regarding the daily diary part of the study, participants received the daily diaries over 21 days (number of observations = 1323), once a day at a 24‐h interval (from 8:00 pm to 12:00 pm). Each day, they reported the intensity of their PEB, for example, how much they recycled, and their EWB, that is, how much they felt connected to others and how much they perceived their life as meaningful (each scale was preceded by the wording: “Think about today…”). The total number of observations was 1282. The retention rate was moderately high, with 68.2% (*n* = 43) of the participants providing data for at least 17 days. In detail, 3.2% (*n* = 2) of participants provided data for seven or fewer days, 14.3% (*n* = 9) answered between eight and 14 daily diaries and 82.5% (*n* = 52) answered between 15 and 21 daily diaries. For a summary of the number of participants who completed the daily diaries each day, please refer to Table 3 available at the OSF link: https://osf.io/bwgcn/?view_only=1a00b7edc18540e6b6d79e325fda2a50.

Concerning the Encouragement part, during the study period each participant received a randomly assigned pro‐environmental encouragement once a day at 8:00 am and received a reminder to check their smartphone at 12:00 pm (i.e., “Good morning. Today we ask you to implement more pro‐environmental actions than you would normally enact on a typical day. Pro‐environmental actions refer to recycling, minimizing energy and plastic consumption, avoiding water waste, or avoiding buying products that have a negative impact on the environment. / Italian translation: Buongiorno. Oggi le chiediamo di mettere in atto più azioni pro‐ambientali di quelle che metterebbe abitualmente in atto in una sua giornata tipo. Per azione pro‐ambientale si intende riciclare, minimizzare il consumo energetico e della plastica, evitare sprechi d'acqua o evitare di acquistare prodotti che hanno un impatto negativo sull'ambiente”). Drawing on the existing literature on encouragement designs (Bradlow 1998; Powers and Swinton 1984; Schmiedek and Neubauer 2020) and considering the relevance of engaging in efforts to perform PEBs for enhancing EWB (Venhoeven et al. 2013), the encouragement message was formulated to solicit small efforts to engage in pro‐environmental actions (i.e., “to implement more pro‐environmental actions than you would normally enact on a typical day”). This approach aimed to assess how these prompted peaks, momentary changes, or deviations in PEBs could influence EWB. Encouragements were sent on 11 non‐consecutive days (around 50% distribution of the 21 measurement occasions). By providing random encouragements to all participants, we were able to randomize occasions (within participants) rather than assigning participants to distinct treatment and control conditions. Moreover, by assessing the impact of the encouraged treatment behavior (i.e., PEB) on a third variable, namely EWB as the outcome, we were able to draw more robust causal conclusions about the treatment effect of encouraged PEB on EWB.

The retention rate was moderately high, with 71.4% (*n* = 45) participants answering at least eight encouragements. Specifically, 28.6% (*n* = 18) of participants answered all 11 encouragement messages. All the analyses were conducted using Mplus 8.11 (Muthén and Muthén 1998‐2019).

### 2.2 Measures

#### 2.2.1 Pro‐environmental behaviors (PEB)

Participants rated their daily PEB using four items aimed at investigating recycling, plastic consumption, energy conservation, and the engagement of others in performing PEBs. The scale was adapted from Menardo et al. (2020) and Kaiser and Wilson (2004). The items were: “Today I recycled”; “Today I limited the consumption of plastic for example, water bottles, products with a lot of packaging”; “Today I limited the consumption of water and electricity”; “Today I encouraged other people to take more care of the environment”. Participants answered on a five‐point Likert scale ranging from 1= “Not at all” to 5 = “Very much”. The four items were averaged. Omega reliability coefficients were 0.53 and 0.85 at the within‐person and between‐person levels, respectively (Geldhof et al. 2014). The Omega coefficient of 0.53 for the within‐person measure is relatively low, which may reflect the heterogeneous nature of PEB, encompassing a diverse set of behaviors (e.g., Lange 2024). This indicates that PEB may be understood as a formative construct, namely a composite of specific component variables (Edwards and Bagozzi 2000). Considering the items as components that together form the construct, internal consistency need not be high (Brose et al. 2020). We selected these items because they represent behaviors that participants could realistically engage in daily, allowing for a reasonable degree of variability in responses over time.

#### 2.2.2 Eudaimonic wellbeing (EWB)

It was assessed through two items aimed at measuring meaning in life (Negri et al. 2020) and closeness to other people (Ryff and Keyes 1995). The items were: “Today I had a good sense of what makes my life meaningful; Today I experienced warm relationships and trust with others”. Participants answered on a five‐point Likert scale (1 = “Strongly disagree”; 5 = “Strongly agree”). The two items were averaged. These items were selected to capture distinct dimensions of EWB: the first reflects a self‐oriented aspect, emphasizing the pursuit of meaningful goals and self‐realization, while the second reflects an other‐oriented aspect, focusing on the importance of fostering positive social connections and contributing to the well‐being of others (Ryan and Deci 2001; Ryff and Keyes 1995). The decision to include only two items was made to balance survey length with participant engagement and data quality, while still measuring essential aspects of EWB.

### 2.3 Statistical Analyses

To infer a causal effect of PEB on EWB, we used a WPED (WPED; Schmiedek and Neubauer 2020) in a DSEM framework (Hamaker et al. 2018). The WPED relies on instrumental variable estimation (e.g., Antonakis et al. 2010; Maydeu‐Olivares et al. 2020) and is employed to establish causality in correlational research or in situations where randomized control trials are not feasible. WPED is a combination of: (a) multilevel modeling to investigate the within‐person relations between variables, (b) the experimental manipulation of a treatment variable at the within‐person level, (c) the use of random encouragements as instrumental variables that allow for dealing with endogeneity issues when a perfect treatment adherence is not possible, which is to be expected in ecologically valid settings (Schmiedek and Neubauer 2020).

In our design, a random encouragement served as a within‐person instrumental variable (z) to isolate the portion of the exogenous variability of the effect of PEB (x) on EWB (y). Following Schmiedek and Neubauer (2020), we evaluated what happened when a person showed a pro‐environmental response (because encouraged to do so) compared to when the same person did not show the same attribute. To use such a design, two conditions are necessary for Instrumental variable estimation (Angrist et al. 1996):

- The random encouragement has a systematic and reliable effect (the stronger the better) on the treatment behavior (adherence effect), which is an empirically testable condition (Schmiedek and Neubauer 2020);
- The effect of the encouragement on the outcome must be fully mediated by the treatment; that is, it must not have a direct effect or any other mediated effect on the outcome (treatment effect), also referred to as the exclusion assumption (Grosz et al. 2024). The second condition cannot be directly tested and can only be supported through theoretical reasoning (Schmiedek and Neubauer 2020).

DSEM is a statistical technique well‐suited to investigate the dynamics of psychological processes utilizing intensive longitudinal data (i.e., data collected at brief intervals, such as hours or days, and with high frequency; McNeish and Hamaker 2020). DSEM is a combination of multilevel modeling, time‐series analysis, and SEM, which allows for the inclusion of latent variables, multiple outcome variables, and mediation effects (Hamaker and Wichers 2017; McNeish and Hamaker 2020). This approach enables the investigation of within‐person dynamics (momentary changes) while controlling for stable between‐person (interindividual) differences (Hamaker et al. 2018).

**FIGURE 1:**
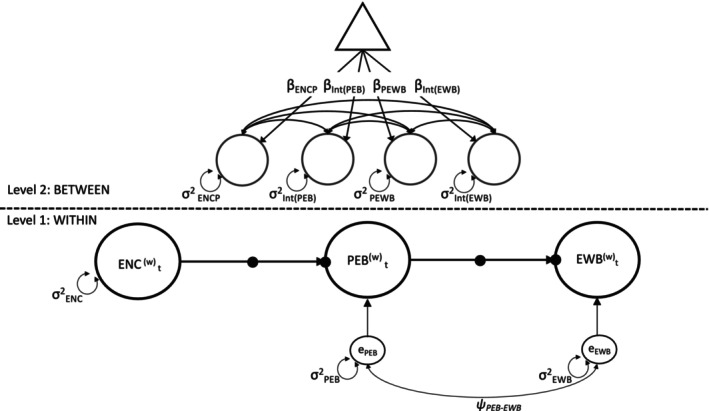
Graphical Representation of the Two‐Level Within‐Person Encouragement Design for the Effect of Pro‐Environmental Behavior (PEB) on Eudaimonic Well‐Being (EWB). On Level 1 of repeated measurement occasions within persons, the path model with direct effects of the instrument (i.e., the encouragement; ENC) on the observed treatment variable (i.e., PEB) and of the treatment on the observed outcome variable (i.e., EWB) is specified. Also, the variances and the covariance (σ^2^_PEB_, σ^2^_EWB_, and *ψ*_PEB‐EWB_) of the residual terms of the treatment (e_PEB_) and the outcome (e_EWB_) at the within‐person level are specified.

In the present study, we used two‐level structural equation modeling to separate within‐person variation (Level 1) from between‐person differences (Level 2), as shown in Figure 1. At Level 1, we estimated a mediation path model with two direct paths from the instrument (encouragement) to the treatment (PEB), and from the treatment to the outcome (EWB). As required by the exclusion assumption, we assumed no direct path from the instrument to the outcome. Moreover, the residual terms of the treatment and the outcome needed to be allowed to correlate with each other, to capture any other (and potentially endogenous) shared influence of the treatment and the outcome that is not due to the encouragement. At the within‐person level, we also included autoregressive parameters from one occasion to the next (Asparouhov et al. 2018), to control for the impact of carry‐over effects of the treatment and outcome variables (Schmiedek and Neubauer 2020). This allowed us to evaluate the extent to which momentary fluctuations (peaks) in PEB or EWB persist over time.

At Level 2, we estimated random effects for intercepts and path regression parameters to allow for individual differences in the baseline frequency of treatment and outcome as well as in the strength of the encouragement‐to‐treatment path and the treatment‐to‐outcome paths.

On Level 2 (between‐person differences), the fixed effects of the encouragement on the treatment (β_ENCP_) and of the treatment on the outcome (β_PEWB_), and the fixed intercepts of the treatment (β_Int(PEB)_) and the outcome (β_Int(EWB)_) are modeled (indicated as paths from the triangle, which represents a constant). Random effects (i.e., between‐person differences) of these effects (σ^2^_ENCP_ and σ^2^_PEWB_) and of the intercepts (σ^2^_Int(PEB)_ and σ^2^_Int(EWB)_), as well as their covariances (double‐headed arrows; parameters not shown) are also included.

Autoregressive effects at Level 1 for PEB and EWB were estimated but are not depicted in the figure.

Following Schmiedek and Neubauer (2020), we performed a model with the following parameters: (a) adherence effect, that is, the within‐person effect of encouragement on PEB; (b) treatment effect, that is, the within‐person effect of PEB on EWB; (c) covariance of the behavior (PEB) and the outcome (EWB) residuals; (d) carry‐over (i.e., autoregressive) effects of PEB and EWB. Encouragement was a dichotomous variable (i.e., encouragement on vs. off), while PEB and EWB were continuous, normally distributed variables.

Given the numerous random effects, Bayesian estimation is the default estimator within the DSEM framework (Hamaker et al. 2018) due to the significant challenges posed by frequentist statistics. It employs Markov Chain Monte Carlo (MCMC) estimation to generate the posterior distribution for all model parameters. To check for model convergence, we relied on the Potential Scale Reduction (PSR) factor and trace plots, and autocorrelation plots from the posterior distribution (Hamaker et al. 2018; Asparouhov and Muthén 2023). A PSR value near 1 indicates that the two default chains estimated by Mplus have converged, while a visual inspection of the trace and autocorrelation plots allows for detecting irregularities (Hamaker et al. 2018).

Adherence, treatment, and carryover effects were modeled as random effects, accounting for their variability among participants. These effects were allowed to covary with each other and with the random intercepts of PEB and EWB at the between‐person level, which reflected participants' overall levels of PEB and EWB across the 21 days.

The analyses were replicated across 1000, 3000, and 5000 iterations to determine which effects maintained statistical significance. We used a thinning factor of 30 to reduce the autocorrelations of the draws from the posterior distributions. In the results section, we exclusively presented effects that remained both stable and statistically significant across the 5000 iterations, while also remaining stable across the models at 1000 and 3000 iterations. To evaluate the statistical significance of these effects, we employed 95% credible intervals (95% CI; McNeish and MacKinnon 2022). Effects were deemed statistically significant if their credible intervals did not contain zero.

## 3 Results

### 3.1 Descriptive Analyses and Bivariate Associations

In Table 1, we report the within‐person correlations between Encouragement, PEB, and EWB; the Intraclass Correlations (ICCs) and the average levels of Encouragement, PEB, and EWB. The latter were obtained by calculating the mean of the participants' scores from the 21 daily diaries. Results showed a positive and significant correlation between ENC and PEB (*r* = 0.088, *p* = 0.003) and between PEB and EWB (*r* = 0.120, *p* < 0.001), while ENC and EWB were non‐significantly correlated (*r* = 0.035, *p* = 0.255).^1^

**TABLE 1:** Within‐Person correlations and intraclass correlations among pro‐environmental behaviors and eudaimonic well‐being.

Variable	*M* (SD)	1	2	3
1. ENC	0.524 (0.499)	(0.000)	0.088*	0.035
2. PEB	3.145 (0.820)	—	(0.762)	0.120**
3. EWB	3.580 (0.779)	—	—	(0.411)

*Note:* The first column shows the means and standard deviations of the variables. Intraclass Correlations (ICCs) are reported on the diagonal.

Abbreviations: ENC, encouragement; EWB, eudaimonic well‐being; M, mean; PEB, pro‐environmental behavior; SD, standard deviation.

* *p* < 0.01.

** *p* < 0.001.

### 3.2 Within‐Person Encouragement Design

The WPED model converged successfully, as indicated by a maximal PSR value of 1.017. Furthermore, a visual inspection of the trace plots and autocorrelation plots revealed no irregularities.

Table 2 depicts standardized and unstandardized parameter estimates of the model. We found a significant positive, yet small, adherence effect of the encouragement on PEB (*β* = 0.093, 95% CI: 0.038, 0.149). The average unstandardized estimate (i.e., the fixed effect) for the adherence effect was *b* = 0.079, 95% CI: 0.015, 0.146, indicating that, on average, encouragements were associated with a 0.079 increase in PEB compared to days without encouragements. For a straightforward quantification of such an effect, considering that the within‐person Standard Deviation (SD)^2^ of PEB was 0.38, the encouragement increased PEB by a factor of 0.21 standard deviations.

**TABLE 2:** Results of the WPED model.

Parameters	Standardized within‐person effects		
	95% CI		
	Estimate	Lower	Upper
Adherence effect			
ENC_t_ → PEB_t_	0.093^a^	0.038	0.149
Treatment effect			
PEB_t_ → EWB_t_	0.227^a^	0.048	0.422
Level 1 residual correlation			
PEB_t_ ↔EWB_t_	−0.123	−0.322	0.063
Carryover effects			
PEB_t‐1_ → PEB_t_	0.307^a^	0.224	0.388
EWB_t‐1_ → EWB_t_	0.155^a^	0.079	0.233

Parameters	Unstandardized fixed effects		
	95% CI		
	Estimate	Lower	Upper
*Fixed effects*			
Intercepts			
PEB	3.083^a^	2.861	3.311
EWB	3.544^a^	3.386	3.702
Adherence effect			
ENC_t_ → PEB_t_	0.079^a^	0.015	0.146
Treatment effect			
PEB_t_ → EWB_t_	0.334^a^	0.055	0.643
Carryover effects			
PEB_t‐1_ → PEB_t_	0.307^a^	0.194	0.421
EWB_t‐1_ → EWB_t_	0.155^a^	0.055	0.253
*Random variances*			
Intercepts			
PEB	0.739^a^	0.492	1.147
EWB	0.325^a^	0.206	0.524
Adherence effect			
ENC_t_ → PEB_t_	0.033^a^	0.012	0.072
Treatment effect			
PEB_t_ → EWB_t_	0.119^a^	0.049	0.253
Carryover effects			
PEB_t‐1_ → PEB_t_	0.085^a^	0.037	0.171
EWB_t‐1_ → EWB_t_	0.053^a^	0.015	0.126

*Note:* Standardized within‐person effect, unstandardized between‐person effects, and random variances.

Abbreviations: CI, 95% credible intervals; ENC, encouragement; EWB, eudaimonic well‐being; PEB, pro‐environmental behavior; WPED, within‐person encouragement design.

^a^ Credible interval did not include zero.

Importantly, we found a significant positive treatment effect of PEB on EWB (*β* = 0.227, 95% CI: 0.048, 0.422). Overall, the model explained 11.7% of the within‐person variance in EWB. Moreover, the correlation among treatment and outcome residuals was nonsignificant (*r* = −0.123, 95% CI: −0.322, 0.063). We also found positive carry‐over effects for PEB (*β* = 0.307, 95% CI: 0.224, 0.388) and EWB (*β* = 0.155, 95% CI: 0.079, 0.233).

At the between‐person level, we found between‐person variability for the path from encouragement to PEB (adherence effect) and from PEB to EWB (treatment effect). Specifically, the random slope variance was 0.033, 95% CI: 0.012, 0.072. As the random slope SD was estimated to be approximately 2.30 times the size of the average fixed effects, this indicates substantial between‐person differences in the adherence effect according to the criteria by Bolger et al. (2019). For the treatment effect, its associated random slope variance was 0.119, 95% CI: 0.049, 0.253. Similar to the adherence effect, the proportion of random slope to fixed effect for the treatment effect was approximately 1.03—again highlighting that individuals differed substantially in the degree to which PEB was causally linked to EWB.

Notably, we found a negative and significant correlation between the adherence effect and the average levels (i.e., the intercept) of PEB (*r* = −0.445, 95% CI: [−0.730, −0.038]), indicating that for participants who reported lower levels of PEB over the 21 days, the effect of the encouragement was stronger.

To explore whether the strength of the adherence and treatment effects rather depended on individuals' general tendency to engage in PEB (i.e., their trait level), we ran an additional model in which we assessed the presence of a cross‐level interaction between a baseline measure of PEBs and the adherence and treatment effects. We did not find any significant interaction, suggesting that the encouragement and the treatment had similar effects on participants, regardless of whether they reported higher or lower baseline levels of PEB. The output of this model is available at the following OSF link: https://osf.io/bwgcn/?view_only=1a00b7edc18540e6b6d79e325fda2a50.

## 4 Discussion

Despite mounting evidence on the positive association of PEBs with EWB (e.g., Zawadzki et al. 2020), existing studies have mostly taken a cross‐sectional approach to the study of such an effect, highlighting the role of stable, interindividual differences (Hamaker et al. 2018). Conversely, the effect of intraindividual changes in PEB performance over EWB remains uncertain and is not corroborated by causal evidence. Taking this into account, emphasizing the immediate benefits of PEBs for individuals in terms of improved well‐being could deepen our understanding of their positive influence on people's daily lives. Moreover, combining experimental methods with ESM (Neubauer et al. 2024) may be of relevance for a better understanding of whether higher‐than‐expected daily levels of PEBs can causally determine higher‐than‐expected daily levels of EWB (Rohrer and Murayama 2023; Zygar‐Hoffmann et al. 2022), particularly in young adults who face developmental as well as climate change –related challenges (Ardoin et al. 2022).

To address these gaps, in the present study we implemented a WPED (Schmiedek and Neubauer 2020) in the context of a daily diary study, which involved a group of young adults followed once a day over 21 days. Specifically, we measured the frequency of PEBs and the intensity of EWB and manipulated PEBs by giving participants random encouragements to engage in more PEBs than usual on 11 days during the study. This method allowed us to test the causal relationship between PEBs and EWB by isolating the portion of PEB variability uniquely responsible for EWB increases (Schmiedek and Neubauer 2020). Moreover, combining it with DSEM allowed us to investigate such an effect by controlling for stable between‐person differences as well as carry‐over effects across days (Hamaker et al. 2018). In other words, we could evaluate whether prompted PEB (treatment) on a given day had a causal positive effect on EWB (outcome) on the same day, despite stable tendencies of individuals to express higher levels of PEB and EWB than others or to respond differently to encouragement and treatment. Through this analytical strategy, we were also able to explore the presence of other within‐person processes such as autoregressive effects, namely whether participants showed persistence in PEBs and EWB over time (Perinelli et al. 2023).

Consistent with prior literature (e.g., Prinzing 2024; Venhoeven et al. 2013; Zawadzki et al. 2020), our study revealed a significant positive causal effect of PEBs on EWB. Specifically, we found that pro‐environmental encouragements predicted positive deviations from individuals' average levels of PEB, corresponding to an increase of approximately 0.21 standard deviations. These increases led to positive deviations from average levels of EWB, with the model explaining about 11.7% of the within‐person variance in EWB. This means that when encouraged, individuals acted more pro‐environmentally than they normally did, and this, in turn, made them experience higher‐than‐usual levels of meaning in life and closeness to other people.

The findings might be interpreted considering the concept of personal agency delineated in the Social‐Cognitive Theory (Bandura 1989, 2001), which refers to individuals' capacity to organize personal resources to pursue goals consistent with their values. Through the exercise of agency, people contribute to personal development by choosing courses of action that reflect such standards and nurture positive self‐views by reflecting on and evaluating their capabilities to pursue such desired goals (Caprara and Steca 2007). Within this perspective, receiving prompts may be considered an opportunity for self‐reflecting on the level of personal involvement in ecological behaviors and on whether and how to engage in behavior change. This in turn can result in enhanced well‐being in terms of experiencing self‐realization through the exercise of one's best qualities (Deci and Ryan 2008). In other words, reflecting on daily personal engagement in PEBs may be an important step for strengthening one's sense of proenviromental agency which, in turn, can have a positive short‐term impact on people's EWB.

Concerning the sample specificity, young adults are in a developmental period in which they have numerous opportunities to put personal resources into play toward the achievement of healthy functioning (Lerner et al. 2015). As suggested by Venhoeven et al. (2013), when individuals deliberately choose to engage in PEBs, and especially when such acts require effort, they experience heightened EWB because they perceive being involved in something meaningful. Thus, for young adults, engaging in more PEBs than usual on certain days may not be a threat to personal well‐being. Rather, it may represent a way to make small changes in one's habits for a good reason that can enrich one's life (Prinzing 2024) and serve as a means to orchestrate personal resources to cope with contextual challenges (Caprara and Steca 2007) such as climate change (Sawitri et al. 2015).

Moreover, PEB engagement can also enhance people's sense of closeness to other people. Specifically, like other forms of prosocial behavior, embracing pro‐environmental actions can strengthen the perception that individuals are acting in the best interests of the community, with such actions being appreciated by society and contributing to the collective welfare (Berger and Andaur 2022; Mac Donald and Staats 2022). Indeed, doing something good for the environment can nurture EWB by fulfilling the basic human needs of agency and communion (Bakan 1966).

From a personality psychology point of view, these results indicated that the impact of PEBs on EWB (i.e., treatment effect) does not only depend on individual (between‐person) differences, but intrapersonal variations from one's typical levels play a role in explaining such an effect. In other words, even individuals who typically engage in PEBs less frequently than others can experience immediate positive effects on EWB when they make efforts to behave more environmentally friendly. This evidence is supported by the presence of a negative and significant association between the average levels of PEB across the 21 days and the adherence effect. This suggests that the effect of the encouragement was stronger for individuals who exhibited lower levels of PEB during the observed period. Additionally, we found no significant cross‐level interaction between trait levels of PEB (i.e., as a baseline measure) and the adherence or treatment effects, suggesting that the strength of the effects did not depend on individuals' typical levels of PEB. Notably, the encouragements had a similar impact on participants, regardless of whether they reported generally engaging in PEBs or not.

These results can have important implications for intervention since they inform on the potential of prompting participants over a short time framework for initiating positive behavior changes as well as on the relevance of increasing individuals' engagement in PEBs in everyday life, to achieve both environmental and health outcomes (Martin et al. 2020). According to the social‐cognitive perspective (Bandura 2001), it could be hypothesized that the encouragements might facilitate participants' self‐reflection on their PEB, thereby enhancing self‐awareness and potentially increasing the likelihood of behavior modification.

Furthermore, our findings showed positive carry‐over effects for both PEBs and EWB. In detail, positive deviations from average levels of PEB/EWB on a day predicted positive deviations of PEB/EWB on the following day. This suggests that, despite stable tendencies, behaving more pro‐environmentally and experiencing higher EWB than usual are likely to endure over time.

At the between‐person level, we found that participants varied in the effectiveness of the adherence and treatment effects. This indicates that in the presence of the encouragement, some individuals showed a higher propensity to behave more pro‐environmentally, while some showed higher levels of EWB due to the treatment (prompted PEB). This result suggests that encouragements can have different effects depending on individuals' characteristics (e.g., Personality traits, values, etc.), information that could be accounted for in future research to design more targeted interventions (Perinelli et al. 2023). For example, research shows that domains of personality such as Openness, Agreeableness and Conscientiousness are positively linked with PEB (e.g., Soutter and Mõttus 2021). More specifically, personality facets such as empathy, cooperativeness, sense of responsibility and generosity are most associated with pro‐environmental behaviors, attitudes and environmental concerns (e.g., Di Fabio and Kenny 2021; Hopwood et al. 2021; Soutter and Mõttus 2021). In terms of values, those reflecting a broader commitment to societal and environmental welfare—such as altruistic and biospheric values—are the most predictive of PEBs (Steg et al. 2014; Steg 2016; Zeiske et al. 2021). These values, which fall under the broader domain of self‐transcendence (Schwartz 1992) not only promote PEBs but also contribute to EWB (Venhoeven et al. 2013). Individuals with strong self‐transcendence values are likely to engage in PEBs out of a genuine commitment to doing good, perceiving their actions as aligned with their values. This experience, in turn, can foster EWB, as it reflects a life lived in accordance with one's fundamental principles (Venhoeven et al. 2013, 2017; Ryan and Deci 2001). Finally, participants differed in their average levels of carryover effects, with some showing a higher persistence of PEBS/EWB on consecutive days. Specifically, they showed a higher propensity than others to engage in PEBs or experience EWB on a day following higher‐than‐usual levels of PEBs or EWB on the previous day.

These results are relevant to inform interventions aimed at increasing PEBs and well‐being, as they show that making everyday changes in PEBs can result in immediate improvements in EWB and that positive changes in PEBs and EWB can persist over time. Thus, supporting individuals' efforts may be beneficial for both longer‐term behavior change and for promoting well‐being, with the potential for greater impact if interpersonal differences are considered in future studies and intervention programs (Perinelli et al. 2023).

## 5 Limitations

The results of the present study should be considered within the context of certain limitations. Firstly, since the adherence effect was positive yet modest, it would be desirable if future studies focus on enhancing adherence to capture longer‐term effects, such as how PEBs influence EWB across consecutive days. Secondly, considering the short‐term impact of PEBs on EWB (i.e., same‐day PEBs predicted same‐day EWB), another limitation could be the time interval used for assessing the variables of interest, that is, the daily diaries. Assessing these variables multiple times per day might better capture a stronger effect. Additionally, employing various time intervals (i.e., hourly or weekly) could enhance the robustness of our findings by examining whether these effects persist, diminish, or disappear entirely. Thirdly, we used one general encouragement about PEBs. Hence, future studies should test whether more specific encouragements, that, for example, address one category of PEBs, can make such a prompt clearer and easier to follow by participants. Moreover, manipulating the dosage of the encouragement could shed light on how to avoid drops in adherence due to habituation (Klasnja et al. 2019).

Additionally, concerning the measurement of daily PEB, it is important to acknowledge some limitations. Unlike well‐established between‐person measurements, there is currently no standardized pool of items available to guide the selection of within‐person measures based on specific theoretical, methodological, and statistical criteria (Brose et al. 2020). Moreover, using a homogeneous set of items, as in a reflective model, may not have been a suitable alternative (Lange 2024) to capture the nuanced variations in individual responses to everyday life events, for example, those stemming from broader personality characteristics (e.g., Soutter and Mõttus 2021). However, employing a heterogeneous set of items, as in a formative measure, while focusing solely on how PEB varied over time in “average” individuals, also has limitations (Brose et al. 2020), as PEB may vary not only over time but also across individuals. To gain a more comprehensive understanding of PEB fluctuations, future studies should explore within‐person variation across both time and individuals, as both factors could influence the reliability estimates of PEB measures (Brose et al. 2020, 2015). Finally, future research should consider other intervening factors that could influence the effect of PEBs on EWB. For example, personality traits (Soutter and Mõttus 2021) and other time‐invariant covariates (e.g., gender, age) may enable the identification of interpersonal differences in the effect of both adherence and treatment effects (Schmiedek and Neubauer 2020), while time‐varying covariates (e.g., life events) could provide a more consistent explanation for the variability in the effectiveness of such an intervention (Zygar‐Hoffmann et al. 2022) in enhancing participants' PEBs and EWB.

## 6 Conclusions

Overall, these research findings suggest that participating in PEBs can lead to non‐materialistic benefits, such as fostering meaning in life and a sense of connectedness to other people. Furthermore, this is particularly true when individuals deliberately choose to engage in PEBs, as it empowers them to attribute significance to their actions and experience enhanced EWB (Venhoeven et al. 2013). From an applied psychology perspective, this could also guide the development of interventions that target situational aspects rather than inherent personality dispositions (Perinelli et al. 2023) in nurturing everyday well‐being through the promotion of pro‐environmental habits. Moreover, from a methodological standpoint, this study seeks to contribute to the understanding of the application of natural experiments in psychology that use instrumental variables (Antonakis et al. 2010; Grosz et al. 2024), specifically in the form of random encouragements within the context of ESM, to draw stronger causal conclusions about the effect of a treatment variable on an outcome. Finally, investigating the impact of PEBs on young people's well‐being in their daily lives could offer insights into how to better support environmentally conscious behaviors among this age group (Carrero et al. 2020) while simultaneously fostering their mental health (Prinzing 2024; Venhoeven et al. 2013). From a Positive Youth Development perspective, which highlights the importance of young people's resources and the plasticity of their developmental pathways (Lerner et al. 2015), encouraging PEBs serves a dual purpose: enabling youth to take an active role in addressing environmental challenges (Bandura 2001; Sanson et al. 2019; Sawitri et al. 2015) and fostering their healthy development (Ardoin et al. 2022). Given that younger generations are particularly vulnerable to the adverse effects of environmental issues on physical and mental health, these behaviors may serve as constructive responses to such challenges (Ojala 2012, 2022).

## Author Contributions

**Silvia Caldaroni:** conceptualization, investigation, data curation, formal analysis, writing – original draft, writing – review and editing. **Maria Gerbino:** conceptualization, investigation, data curation, writing – original draft, writing – review and editing. **Florian Schmiedek:** conceptualization, formal analysis, writing – review and editing. **Andreas B. Neubauer:** conceptualization, formal analysis, writing – review and editing. **Lucia Manfredi:** investigation, data curation, writing – review and editing. **Fulvio Gregori:** investigation, data curation, writing – review and editing. **Concetta Pastorelli:** conceptualization, investigation, writing – review and editing. **Giuseppe Corbelli:** formal analysis, writing – original draft, writing – review and editing. **Antonio Zuffianò:** conceptualization, investigation, data curation, formal analysis, writing – original draft, writing – review and editing.

## Supporting information


**Data S1.** Supporting Information.

## Data Availability

Data and analysis scripts used for this article can be accessed at the following link: https://osf.io/bwgcn/?view_only=1a00b7edc18540e6b6d79e325fda2a50.

## Endnotes

^1^
The two items measuring EWB (meaning in life and connectedness with others) were significantly and positively correlated, *r* = 0.492, *p* < 0.001.

^2^
The within‐person SD for PEB was calculated as the square root of the Level 1 residual variance derived from a two‐level model with PEB as the dependent variable and no predictors. The model is reported in the Supplemental materials available at the OSF link: https://osf.io/bwgcn/?view_only=1a00b7edc18540e6b6d79e325fda2a50.
