# Supplementary material for: The Positive Effect of pro‐Environmental Behavior on Eudaimonic Well‐Being in Young Adults: A Daily Diary Study Using the Within‐Person Encouragement Design
Source: J Pers. 2025 Mar 20;94(1):124–36. doi: 10.1111/jopy.13021 (PMC12780324; doi:10.1111/jopy.13021)
Supplement: Supplementary file 1 — Data S1. Supporting Information. [file JOPY-94-124-s001.zip › Codebook.docx]

**Codebook of the Daily Diary Study**

This document presents the daily encouragement participants received on 11 of the 21 days of the study, as well as the measurements completed during the 21-day period aimed at evaluating their pro-environmental behaviors and eudaimonic well-being. Only the items relevant to the present study are reported, but the study was part of a larger longitudinal research project exploring the role of prosocial behavior, emotional regulation, and pro-environmental behavior in fostering young adults' well-being in daily life. As a result, additional measures were collected, including positive and negative affect, and prosocial behaviors.

**Encouragement message**

(It was sent at 8:00 am on 11 random days, with a reminder at 12:00 pm).

Good morning. Today we ask you to implement more pro-environmental actions than you would normally enact on a typical day. Pro-environmental actions refer to recycling, minimizing energy and plastic consumption, avoiding water waste, or avoiding buying products that have a negative impact on the environment.

Italian translation: Buongiorno. Oggi le chiediamo di mettere in atto più azioni pro-ambientali di quelle che metterebbe abitualmente in atto in una sua giornata tipo. Per azione pro-ambientale si intende riciclare, minimizzare il consumo energetico e della plastica, evitare sprechi d’acqua o evitare di acquistare prodotti che hanno un impatto negativo sull’ambiente.

**Daily diary**

(The survey was sent at 8:00 pm every day. Participants could answer until 12:00 am. The measurements focus on four daily pro-environmental behaviors, as well as experiences of meaning in life and closeness to other people).

**Pro-environmental behaviors**

Think about today and indicate how much you have engaged in the following behaviors:

*(1) Not at all – (5) Very much*

1. Today I recycled;
2. Today I limited the consumption of plastic (for example, water bottles, products with a lot of packaging;
3. Today I limited the consumption of water and electricity;
4. Today I encouraged other people to take more care of the environment.

Italian translation: Pensi ad oggi e indichi quanto ha messo in atto i seguenti comportamenti:

*(1) Per nulla – (5) Moltissimo*

1. Ho fatto la raccolta differenziata;
2. Ho limitato il consumo di plastica (per esempio, bottigliette d’acqua, prodotti con tanto imballaggio);
3. Ho limitato il consumo di acqua ed energia elettrica;
4. Ho spronato altre persone a prendersi più cura dell’ambiente.

**Eudaimonic well-being**

Think about today and express your level of agreement/disagreement with the following statements.

*(1) Strongly disagree – (5) Strongly agree*

1. Today I had a good sense of what makes my life meaningful;
2. Today I experienced warm relationships and trust with others.

Italian translation: Pensi ad oggi ed esprima il suo grado di accordo/disaccordo con le seguenti affermazioni.

*(1) Fortemente in disaccordo – (5) Fortemente d’accordo*

1. Oggi ho avuto una buona percezione di ciò che rende la mia vita significativa;
2. Oggi ho sperimentato relazioni calorose e di fiducia con gli altri.
